# Chlorinated Pools and the Risk of Asthma

**DOI:** 10.1289/ehp.115-a240a

**Published:** 2007-05

**Authors:** Peyton A. Eggleston

**Affiliations:** Johns Hopkins University, Baltimore, Maryland, E-mail: pegglest@jhmi.edu

In a recent article, Bernard et al. (2006) presented data that led them to conclude that the use of chlorinated pools, especially by young children, interacts with atopic status to promote the development of childhood asthma. I question these conclusions for several reasons.

First, this finding is not consistent with the authors’ recent publication from this same group of children (Nickmilder et al. 2005) concluding that children living in a home cleaned with chlorine bleach had a lower prevalence of asthma. It is difficult to understand how occasional exposure to chlorinated compounds at indoor swimming pools could cause asthma if more frequent and longer exposures at home were actually protective.

Second, the data presented by Bernard et al. (2006) do not fully support their conclusion. For example, the exposure metric they used to describe the children’s exposure to chlorinated pools is the lifetime cumulative swimming pool attendance (CPA) given in hours. The CPA data are based on lifetime exposure derived from questionnaires that the parents of these 11- to 12-year-old children completed at home [American Thoracic Society, European Respiratory Society (ATS/ERS) 2005] and is thus subject to their understanding and interpreting the question, as well as to recall bias. In addition, systematic bias is introduced by using a lifetime cumulative measure like CPA to relate exposure to asthma prevalence. Lifetime cumulative exposure is obviously dependent on the age of the child; because asthma prevalence also increases during this same time, the child’s age becomes a confounder that cannot be dealt with adequately in the analysis used by Bernard et al. (2006).

Third, the data presented to relate the dose response between CPA and asthma prevalence are confusing. In Table 2 of Bernard et al. (2006), the relationship is not significant, while in Figure 1 it is significant in a subgroup. In their Figure 1A, a dose response is suggested between CPA and the prevalence of doctor-diagnosed and total asthma, but only in those children whose total IgE is > 100 IU/mL. The subgroups in this figure are small; from data in Table 1 and the text, it appears that only 14 children had both IgE > 100 IU/mL and doctor-diagnosed asthma, and only 20 had total asthma with a high IgE concentration. Because Figure 1 (Bernard et al. 2006) divides all 341 children into approximately equal quartiles of CPA, it seems impossible to allocate the 14–20 children with asthma in such a way that would result in an asthma prevalence of 12–35% within each quartile. I suggest that the figure is drawn incorrectly and that the correct relationship is shown in their Table 2.

Fourth, insufficient information is available to address the uncertainties in the outcome measures of Bernard et al. (2006). The data in their Table 2 demonstrate that swimming pool attendance was associated with the prevalence of an elevated exhaled nitric oxide (eNO); neither doctor-diagnosed asthma nor total asthma was significantly related to swimming pool attendance unless combined with eNO measures. Although eNO is associated with asthma, it has been used primarily to measure the state of airway inflammation in asthma; the use of eNO is less certain as a diagnostic tool (ATS/ERS 2005). In fact, elevated eNO levels have been associated with viral respiratory tract infections, allergic rhinitis, and sinusitis (ATS/ERS 2005). These conditions were not included in the health questionnaire described by Bernard et al. (2006) in their “Materials and Methods.” Indeed, only 20 of the 29 children with an elevated eNO (> 30 ppb) had doctor-diagnosed asthma. In addition, the study was conducted during winter months when viral respiratory infections are common; therefore, the presence of these infections could have produced outcome misclassification. Finally, inhaled steroid medications markedly reduce eNO, and use by these children could have introduced yet another reason for outcome misclassification.

To summarize, the uncertainty in both the exposure estimates and the outcome measures, coupled with the conflicting outcomes with home exposure to chlorine bleach, make it difficult to accept the strong conclusions reached by Bernard et al. (2006) in their article.
